# Retrospective Review Identifying Patients With Bacteremia and Intracardiac Devices With an Electronic Health Record Advisory

**DOI:** 10.7759/cureus.74012

**Published:** 2024-11-19

**Authors:** Gabriel Velez Oquendo, Riaz Mahmood, Joon Ahn, Shane Robinson

**Affiliations:** 1 Internal Medicine, Northeast Georgia Medical Center Gainesville, Gainesville, USA; 2 Electrophysiology, Northeast Georgia Medical Center Gainesville, Gainesville, USA; 3 Research, Northeast Georgia Medical Center Gainesville, Gainesville, USA

**Keywords:** bacteremia, cardiac device infection, cardiac implantable electronic device (cied), pacemaker, pacemaker infection

## Abstract

Background: Cardiovascular implantable electronic device (CIED) infections without early diagnosis, treatment, and proper follow-up are associated with increased morbidity, mortality, and worse outcomes.

Objective: This study aims to identify patients presenting for hospital admission with bacteremia and the presence of CIED by implementing a best practice advisory (BPA) notification in the electronic medical record to facilitate early consultation with the cardiac electrophysiology (EP) team and treatment.

Methods: A BPA was implemented into the electronic medical record (EMR) EPIC in 2022 and was generated for any patient that presented to our health system with bacteremia and the presence of a CIED. The BPA gave the provider an option for EP consultation. Data was collected from EPIC from 2021 to 2023 using the International Classification of Diseases, Tenth Revision, Procedure Coding System (ICD10-CM/PCS) codes to identify patients and comorbidities. A comparative analysis was conducted to determine the effectiveness of the BPA in increasing EP consults and cardiac device extraction procedures, as well as overall outcomes.

Results: A total of 447 patients were diagnosed with bacteremia and the presence of a CIED during the study period, with 178 before the BPA and 269 status post-BPA. The BPA resulted in a nonsignificant increase in EP consultations from 19.66% to 25.88% (p = 0.168) and device extractions from 9.55% to 13.75% (p = 0.182). EP consults were a significant predictor for device extractions (odds ratio (OR) = 9.4644, p < 0.0001). The mortality rate decreased from 17.42% to 14.13% (p = 0.419), and the 30-day readmission decreased from 14.37% to 12.41% (p = 0.652).

Conclusion: While the BPA did not show significant improvements, its implementation shows promise over time with positive trends in consults, extractions, and in-hospital mortality.

## Introduction

Approximately three million patients in the United States have permanent pacemakers, and more than 300,000 of these patients have an implantable cardioverter defibrillator (ICD). [1] Although the use of cardiac implantable electronic devices is linked to improved clinical outcomes and reduced mortality, they are also associated with complications, including lead-related complications, device malfunction, and infections, which are increasingly prevalent worldwide [2]. The incidence of infection in patients with cardiac devices has been difficult to determine due to the absence of a comprehensive registry. Nevertheless, observational studies have demonstrated a 1.2% to 3.4% risk of developing device infection [3]. This is associated with high morbidity and mortality in up to 10%-30% of patients experiencing infection and occurs more frequently in patients with systemic infections or bacteremia, which amounts to 52.8% as the most common indication for complete device extraction according to the “European lead extraction controlled registry” [4,5].

Increasing placement procedure rates of cardiac devices in older patients with multiple comorbidities have set the stage for higher rates of device-related infections [6]. Debates in the literature, including a meta-analysis of 60 prospective and retrospective studies from the European Society of Cardiology (ESC), highlight that risk factors such as diabetes mellitus, end-stage renal disease, heart failure, and chronic obstructive pulmonary disease are more prevalent in this population, further contributing to the risk of infections [6,7]. Cardiac device-related infections represent a significant risk, especially in patients with preexisting comorbidities [7]. Analysis from the United States Renal Data System indicates that a notable proportion of patients with cardiac devices develop infections, with only a fraction of infected devices being removed [6]. Moreover, a substantial percentage of patients with cardiac devices who develop bacteremia are at risk of developing a device-related infection [8].

The American Heart Association (AHA) guidelines recommend complete device removal due to the high rate of infection relapse with retention of a device, with one study on the early diagnosis of device infection and early lead extraction within three days of diagnosis being associated with lower in-hospital mortality, shorter length of stay (LOS), and increased overall survival [9,10]. Therefore, when a patient with a cardiac device is diagnosed with bacteremia, a consultation is essential to assess the device and determine if extraction, along with appropriate antibiotic therapy, is necessary after thorough evaluation, potentially employing specific imaging techniques in clinically inconsistent cases. However, device removal is often delayed in favor of initial trials of antimicrobial therapy in clinical practice [11]. In essence, when a patient who has a cardiac device in situ gets diagnosed with bacteremia, a consult with the cardiac electrophysiology (EP) should take place for evaluation of the device and possible device infection. During evaluation, if there is a device infection in the setting of bacteremia, device extraction should take place along with antibiotic management. According to a survey from the European Heart Rhythm Association, over 50% of physicians felt they lacked the knowledge and skills to make the diagnosis and refer for lead extraction, and over 75% felt they lacked knowledge and skills to manage aspects of extraction and post-extraction care [12]. They found significant gaps in physicians' knowledge and skills.

Communication and reminders of guideline-directed patient care for these patients are needed to help ensure proper assessment and management of patients with bacteremia to exclude and/or diagnose cardiac device infection. A best practice advisory (BPA) in the electronic medical record (EMR) serves as a way to improve physician awareness and identify all patients admitted with bacteremia who also have an intracardiac device as it serves as a reminder that the cardiac EP service should be consulted for patients who have cardiac device infections for evaluation of extraction to reduce mortality and hospitalization rates. 

Our study seeks to assess the effectiveness of implementing a BPA at our center to enhance the identification of patients with bacteremia and a cardiovascular implantable electronic device (CIED). We will also evaluate the impact on the number of consultations with the cardiac EP service for device extraction in patients who developed bacteremia. This comparison will be made for the year preceding and the year following the implementation of the BPA notification in the institution's EMR (EPIC).

## Materials and methods

Data source

**Figure 1:**
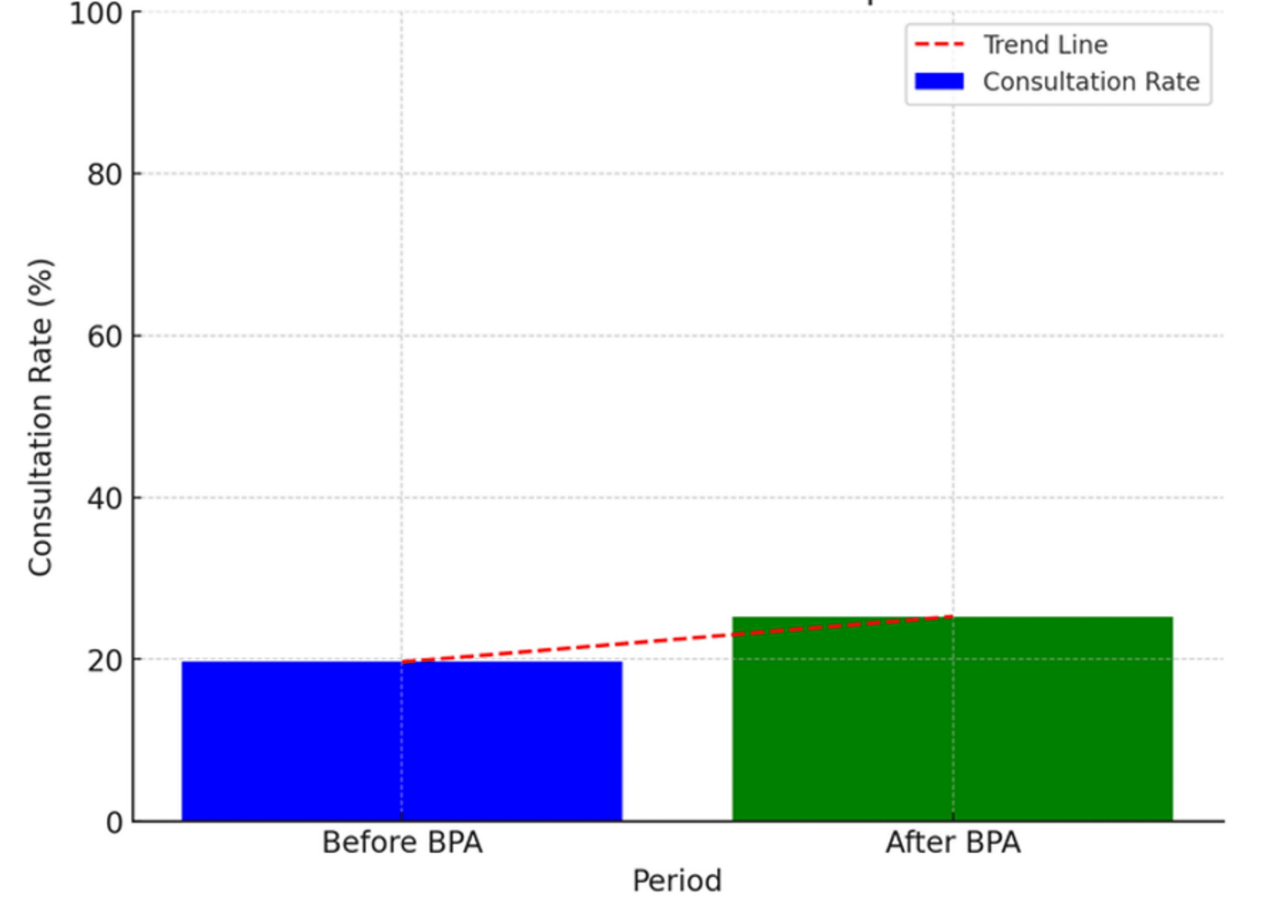
This graph demonstrates a noticeable increase in the consultation rate post-BPA. The trend line emphasizes the upward shift, showing that the BPA contributed to more frequent consultations. This suggests that the advisory may have improved awareness and the process for consulting the cardiac electrophysiology service

Data was obtained from a single institution’s EPIC electronic medical health records (EMHR) at Northeast Georgia Medical Center from 2021 to 2023, one year before and after the “Best Practices Advisory” was implemented in 2022. The BPA allowed the provider to consult EP when patients were diagnosed with bacteremia (Figure 1). The database contains deidentified information from 447 patients diagnosed with bacteremia from the healthcare system by the blinded GME data administrator. This data was not publicly available, so this study was granted expedited ethical approval from the Institutional Review Board at Brenau University in Gainesville, Georgia. 

Study population

The International Classification of Diseases, Tenth Revision, Procedure Coding System (ICD10-CM/PCS) codes were used to identify adult patients with intracardiac devices afflicted with bacteremia and device extraction procedures. We differentiated patients with pacemakers versus intracardiac defibrillators with ICD-10 codes Z95.0 and Z95.810, respectively. Furthermore, we used CIED current procedural terminology (CPT) codes 33234, 33235, 33244, and 33249 to represent cardiac device extractions. 

Definition of variables

We used EPIC chart variables to extract data regarding the age, gender, race, ethnicity, and medical history of the patients with bacteremia. Variables, including comorbidities like coronary artery disease, congestive heart failure, diabetes mellitus type 2, obesity, and obstructive sleep apnea, were identified using ICD-10 codes. Furthermore, these codes were used to identify device types and bacteremia. Inpatient admission hospitalization types were the only ones included in this study. This data was validated by the author and the research team, which included two independent reviewers.

Outcomes

The primary outcomes assessed were the frequency of consultations with the cardiac EP service that resulted in cardiac device extractions, along with any changes in this frequency over the year following BPA implementation. Secondary outcomes included hospital mortality and inpatient LOS. Additionally, we evaluated the influence of age, gender, race, ethnicity, and risk factors for CIED infection or bacteremia on the rate of device extractions. 

Statistical analysis

All statistical analyses were performed with JMP Pro 17 statistical software (SAS Institute Inc., Cary, NC). Descriptive analyses were used to characterize the patients identified in both timeframes before and after BPA implementation. Then, parametric independent T-tests were utilized to determine the effectiveness of the BPA by evaluating the rates of consults, mortality, and hospital LOS. Next, the clinical characteristics of patients with pacemakers and implantable cardiac defibrillators (ICDs) were compared between both groups regarding the implementation of the BPA with a logistics regression model. Furthermore, using a multivariable logistic regression model, we elucidated the factors associated with greater odds of receiving a consultation with the cardiac EP service and receiving a device extraction. They were also used to determine if there were causation between variables. An alpha level of p < 0.05 was set for all statistical tests. 

## Results

A total of 447 patients were diagnosed with bacteremia between 2021 and 2023, and most of these patients were in the status post-BPA group (n = 269) as compared to 178 patients in the pre-BPA group. In the pre-BPA group, most of the patients were males (57.3%, n = 102), White (37.5%, n = 168), non-Hispanic (39.3%, n = 176), and with a mean age of 66.99 +- 12.79 years of age. In comparison, in the BPA group, most of these patients were males (62.5%, n = 168), White (54.5%, n = 244), and non-Hispanic (57.7%, n = 258), with a mean of 67.39 ± 12.24 years of age. In terms of cardiometabolic diseases, the post-BPA group had more patients with coronary artery disease, congestive heart failure, hypertension, and type 2 diabetes mellitus, but there were no statistically significant differences (Table 1).

**Table 1:** Demographic characteristics and comorbidities of patients pre-BPA and post-BPA BPA: Best practice advisory p < 0.05 was considered statistically significant

	Pre-BPA	Post-BPA	p-value
Total: 447 patients	178	269	<1.000
Age			
18-34 years	3	1	<0.3519
35-49 years	2	6	<0.6172
50-64 years	29	47	<0.8442
65-79 years	78	110	<0.6058
>80 years	66	105	<0.7513
Male	102	168	<0.3216
Female	76	101	<0.3216
Race			
White	168	244	<0.2163
Black	0	0	<1.000
Hispanic	0	0	<1.000
Asian	1	0	<0.3982
Other	9	25	<0.1409
Ethnicity			
Not Hispanic, Latino/a, or Spanish origin	176	258	<0.1237
Hispanic, Latino/a, or Spanish origin	2	5	<0.8229
Hispanic or Latino/a	0	0	<1.000
Declined to answer	0	0	<1.000
Other	0	6	<0.0855
Comorbidities			
Coronary artery disease	102	149	<0.6898
Congestive heart failure	141	216	<0.7799
Hypertension	89	133	<0.9081
Diabetes mellitus type 2	86	133	<0.8154

**Figure 2:**
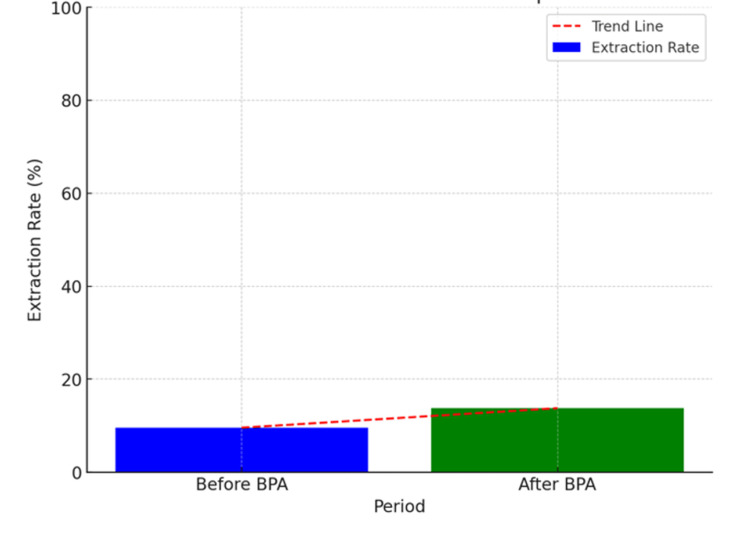
This comparison illustrates an increase in the rate of device extractions after the BPA was introduced BPA: Best practice advisory

During the study period between 2021 and 2023, out of the 447 patients with bacteremia, the BPA implementation led to an increase in consultations from 19.7% to 25.3%. Of these consultations (p = 0.168), 54 led to cardiac device extraction secondary to bacteremia, with 9.6% pre-BPA and 13.8% post-BPA with a p-value = 0.225 (Figure 2). The demographic characteristics and comorbidities of patients who had a consultation and underwent cardiac device extractions in the setting of bacteremia are shown (Tables 1-2). The odds ratio (OR) of having an EP consult if the patient had a pacemaker was 1.490 (95% CI: 0.800-2.775; p-value of 0.209) compared to patients with an intracardiac defibrillator. 

**Table 2:** Outcomes of patients consulted pre-BPA and post-BPA for evaluation of cardiac device extraction BPA: Best practice advisory p-value < 0.05 was considered statistically significant

	Pre-BPA	Post-BPA	p-values
Total consults	35	68	<0.1646
Extraction	17	37	<0.1763
LOS (median days)	8.30	9.19	<0.4016
Mortality	31	38	<0.419
Readmission at 30 days	25	33	<0.6002
Readmission at 60 days	35	48	<0.6210
Readmission at 90 days	39	61	<0.6546

**Figure 3:**
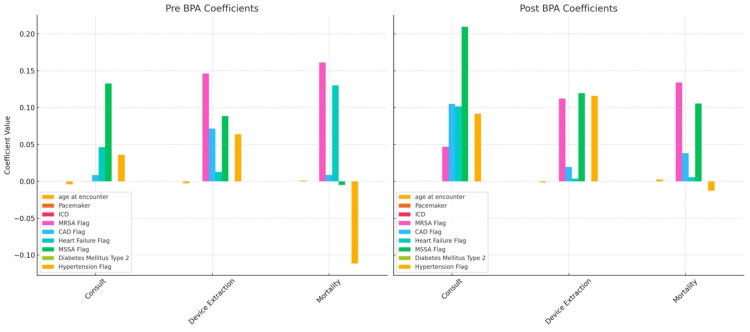
This graph compares the influence of various patient characteristics and clinical factors on three outcomes: consultations, device extractions, and mortality. Each bar in the graphs represents the coefficient of a variable from a regression model, which estimates the effect of that variable on the outcome. The graphs are split to show the coefficients before (pre-BPA) and after (post-BPA) the implementation of the best practice advisory (BPA)

Before BPA implementation, the cardiac device extraction rate was approximately 9.55% (95% CI: 5.23-13.87) compared to 13.75% (95% CI: 9.64-17.87, p-value < 0.1763) (Table 2, Figure 3). The OR of having a cardiac device extraction after a consult was placed was 9.4644 (95% CI: 4.7744-18.7614, p-value < 0.0001). The comorbidities associated with a higher likelihood of getting a consult were heart failure with an OR of 2.7699 (95% CI: 1.2899-5.9480, p-value = 0.0090) and hypertension with an OR of 1.7483 (95% CI: 1.0405-2.9377, p-value = 0.0349). Factors that affected the rate of device extraction included age, with a mean age of 66.85, sex with males comprising 50.4%, and those affected with coronary artery disease and hypertension. Moreover, factors increasing mortality included those affected with heart failure, hypertension, diabetes mellitus type 2, and infection with methicillin-resistant *Staphylococcus aureus *(MRSA), with patients being mostly male (58.3%) and a median age of 67.6 (Figure 4). 

The median LOS pre-BPA was 8.30 days (95% CI:10.65-13.91) compared to the post-BPA, which was 9.19 days (95% CI: 11.01-21.67) with a p-value of <0.4016. Furthermore, the mortality rate pre-BPA was 17.42% (95% CI: 12.55-23.66) to 14.13% post-BPA (95% CI: 10.47-18.79) with a p-value of 0.419. Additionally, the OR for mortality after a consultation was 0.9738 (95% CI: 0.4827-1.9644, p-value < 0.9410). All these p-values were not statistically significant. Ultimately, the readmission rate within 30 days pre-BPA was 14.04% (95% CI: 9.93, 20.35) compared to 12.27% post-BPA (95% CI: 8.97% to 16.91%) with a p-value of <0.6002, and this was evaluated with an electronic database assessment. The p-value indicated that there were no statistically significant differences in readmission rates. 

## Discussion

This study assessed the impact of implementing a BPA within the EPIC EMHR system on the number of consultations with the cardiac EP service. The aim was to enhance the identification of patients who have bacteremia with suspected CIED infection who might benefit from cardiac device extraction at our institution. After BPA implementation, EP consultation rates rose from 19.66% to 25.28%, though this increase was not statistically significant (p = 0.1646). Among patients who received EP consultations, there was a significant rise in device extraction procedures, with an OR of 9.4644 (p < 0.0001), consistent with the institution's requirement for EP consultation before any device or lead extraction. The majority of patients did not undergo device extraction for reasons such as blood culture contaminants, advanced age and frailty, and comorbidities. Additionally, some of the patients had other identified focus of infection responsible for the bacteremia, and that’s the reason they were not proposed for device extraction.

We were also able to determine the different factors that affected the number of consultations, device extraction, and mortality. These factors included heart failure, hypertension, diabetes mellitus type 2, and coronary artery disease, which could be related to socioeconomic factors. Unfortunately, despite the positive trends in the number of consultations and device extraction, the mortality rate and LOS did not show statistically significant improvements in the entire cohort. This lack of significance in mortality reduction could be attributed to other factors beyond the BPA, such as the severity of comorbidities in the patient population, as described above. Finally, while the BPA did not lead to statistically significant improvements in outcomes, the trends indicate a promising direction that, over time, could yield more impactful results as the healthcare team becomes more accustomed to the BPA prompts and integrates this practice into their routine care.

These results concur with some studies, with one showing that CIED infections secondary to bacteremia carry high short- and long-term mortality risk, with some studies showing an estimated one in five patient deaths occurring within one year and a 50% risk of mortality at three years, emphasizing the importance of timely intervention in patients with cardiac devices and bacteremia [13]. This study also showed that quality of life was significantly reduced at the time of infection diagnosis as compared with baseline, and it did not return to normal levels 6 months after diagnosis [13].

It is important to know that in the setting of CIED infection, antibiotics alone are not effective as a lone treatment and can have significant adverse consequences, including higher rates of infection relapse and mortality [11]. Complete device and lead removal in patients with definite CIED infection from bacteremia or infective endocarditis is standard of care and carries a class I recommendation in clinical practice guidelines. Studies have shown that extractions performed earlier after diagnosis are associated with significantly lower one-year mortality rates as well as shorter lengths of hospital stay and fewer physician or hospital visits, which is what our study aimed to improve [11].

One study that included 416 patients from 1991 to 2008 found that immediate removal of CIED was associated with lower one-year mortality (HR, 0.35; 95% CI, 0.16-0.75) [14]. Additionally, a second analysis that included 12,999 patients with CIED infection between 2016 and 2018 found that in-hospital mortality was higher when extraction was delayed more than seven days (HR, 1.55; 95% CI, 1.29-1.87) [15]. These and our study reinforce the notion that systematic approaches, such as the implementation of BPAs, can enhance clinical practice by increasing awareness and facilitating timely consultations. The implementation of BPAs in EMHR systems can play a vital role in improving the management of patients with cardiac device-related infections. The observed increase in consultations and device extractions after only one year following implementation suggests that BPAs can effectively prompt healthcare providers to take necessary actions, potentially leading to better patient outcomes. 

This study has several limitations. First, the retrospective nature of the analysis may introduce biases related to data collection and reporting. Second, the study was conducted in a single healthcare system, which may limit the generalizability of the findings. Third, the relatively small sample size and the lack of statistically significant changes in some outcome measures call for larger, multicenter studies to validate these findings and assess the long-term impact of BPAs.

Future studies can focus on conducting larger-scale, multicenter studies to evaluate the effectiveness of BPAs in various healthcare settings. Additionally, exploring the integration of BPAs with other clinical decision-support tools could further enhance their utility and impact. Furthermore, investigating the reasons for delays in device extraction and developing targeted interventions to address these barriers can also improve patient outcomes.

## Conclusions

The risk of serious cardiac implantable device-related infections in the setting of bacteremia continues to increase for each device type. Analysis revealed that the implementation of an advisory in the EMHR that alerts physicians about consultation to the cardiac EP service for patients with cardiac devices who are afflicted with bacteremia not only increases the number of consults to the service but also increases the rate of cardiac device extraction. The increase in device extraction led to an increase in the hospital LOS, likely associated with delays in procedure room availability. Furthermore, there was a decrease in the rate of readmission.
